# Supplementary material for: Resolving the full spectrum of human genome variation using Linked-Reads
Source: Genome Res. 2019 Apr;29(4):635–45. doi: 10.1101/gr.234443.118 (PMC6442396; doi:10.1101/gr.234443.118)
Supplement: Supplemental Material [file supp_gr.234443.118_Supplemental_longranger-2.2.zip › longranger-2.2.tar.bz2!/lr2.2-public/lib/stan/cmdstan/stan/doc/api/html/check__size__match_8hpp_source.html]

Stan: src/stan/math/error\_handling/matrix/check\_size\_match.hpp Source File


|  |  |
| --- | --- |
|  | Stan  2.5.0  probability, sampling & optimization |


- Main Page
- Related Pages
- Namespaces
- Classes
- Files

- File List
- File Members

All Classes Namespaces Files Functions Variables Typedefs Enumerations Enumerator Friends Macros Pages

- src
- stan
- math
- error\_handling
- matrix

check\_size\_match.hpp

Go to the documentation of this file.

1 #ifndef STAN\_\_MATH\_\_ERROR\_HANDLING\_\_MATRIX\_\_CHECK\_SIZE\_MATCH\_HPP

2 #define STAN\_\_MATH\_\_ERROR\_HANDLING\_\_MATRIX\_\_CHECK\_SIZE\_MATCH\_HPP

3

4 #include <sstream>

5 #include <boost/type\_traits/common\_type.hpp>

6 #include <stan/math/error\_handling/dom\_err.hpp>

7

8 namespace stan {

9  namespace math {

10

11  // FIXME: update warnings

12  template <typename T\_size1, typename T\_size2, typename T\_result>

13  inline bool check\_size\_match(const char\* function,

14  T\_size1 i,

15  const char\* name\_i,

16  T\_size2 j,

17  const char\* name\_j,

18  T\_result\* result) {

19  typedef typename boost::common\_type<T\_size1,T\_size2>::type common\_type;

20  if (static\_cast<common\_type>(i) == static\_cast<common\_type>(j))

21  return true;

22

23  std::ostringstream msg;

24  msg << name\_i << " (%1%) and "

25  << name\_j << " (" << j << ") must match in size";

26  std::string tmp(msg.str());

27  return dom\_err(function,i,name\_i,

28  tmp.c\_str(),"",

29  result);

30  }

31

32  }

33 }

34 #endif

dom\_err.hpp

stan::math::dom\_err

bool dom\_err(const char \*function, const T &y, const char \*name, const char \*error\_msg, const T\_msg error\_msg2, T\_result \*result)

**Definition:** dom\_err.hpp:36

stan::math::check\_size\_match

bool check\_size\_match(const char \*function, T\_size1 i, const char \*name\_i, T\_size2 j, const char \*name\_j, T\_result \*result)

**Definition:** check\_size\_match.hpp:13

stan::math::common\_type

**Definition:** common\_type.hpp:13


---

[ Stan Home Page ]

*© 2011–2014,
Stan Development Team.*
